# Evaluation of the degree of knowledge of gingival melanosis between professionals and students: observational and cross-sectional study

**DOI:** 10.3389/froh.2025.1707785

**Published:** 2025-11-28

**Authors:** María Lourdes Alfaro-Ochoa, Aurore Duplin, Santiago Arias-Herrera

**Affiliations:** Faculty of Health Sciences, Department of Dentistry, Universidad Europea de Valencia, Valencia, Spain

**Keywords:** gingival melanosis, diagnosis of gingival melanosis, treatment of gingival melanosis, survey, dentists' and students' dental knowledge, knowledge and attitudes of oral health professionals

## Abstract

**Introduction:**

Gingival melanosis is characterized by a change in the physiological color of the gingiva, that usually ranges from brown to black. It can be a source of discomfort for affected people, an attractive gingiva being associated with a pink color. The main objective of this study was to compare knowledge about gingival melanosis between fourth- and fifth-year dental students and dental professors at the European University of Valencia.

**Methods:**

The conduct of this cross-sectional observational study was approved by the Research Ethics Committee of the European University of Valencia. The survey used was drafted by the authors of the study. Participants completed the questionnaire between February and March 2024. Statistical analysis was performed using IBM SPSS 23.0.

**Results:**

Significant differences were found between the knowledge of both groups regarding monthly and annual detection frequency (*p* < 0.001), diagnostic with the DOPI index (*p* = 0.048), treatment options (*p* < 0.001), recurrence (*p* = 0.004) and impact on the patient's life (*p* = 0.012). Professional knowledge was higher, except regarding the impact. Conversely, there were no significant differences in the detection of gingival melanosis, etiologies, the ways to transmit information to the patient and diagnosis using the melanin and gingival pigmentation indices.

**Conclusions:**

It can be concluded that teachers’ knowledge is more important in terms of detection frequency, use of the DOPI index, treatment options and recurrence, except for the impact on the patient's life. However, in terms of detecting gingival melanosis in the oral cavity, possible etiologies and diagnostic methods such as the melanin and gingival pigmentation index, knowledge is the same between both groups.

## Introduction

An attractive smile is represented, in part, by having healthy gingiva. This includes a suitable color, position and shape (1, 2). Gingival melanosis is characterized as an alteration that triggers a color change in the gingiva, caused by the accumulation of melanin pigments within the basal and suprabasal layers of the gingival epithelium (3, 4). It can be a source of discomfort for affected people, as a pink gingival color is culturally associated with oral health and esthetics (3–7). Gingival pigmentation affects all ethnicities and genders (5, 6) and may appear either physiologically or pathologically (2, 3, 6, 7). Physiological melanosis is benign and related to genetic and racial factors, whereas pathological pigmentation can be secondary to systemic diseases (such as Addison's disease or Peutz–Jeghers syndrome), certain medications (antimalarials, minocycline, chemotherapeutic agents), smoking, or malignant conditions such as oral melanoma (8, 9). Therefore, accurate clinical diagnosis is essential to differentiate physiological pigmentation from lesions with pathological potential.

Diagnosis of gingival melanosis is mainly clinical and based on visual inspection under adequate lighting. Several indices have been proposed to standardize its assessment, including the Dummett–Gupta Oral Pigmentation Index (DOPI) and the Gingival Melanin Index (GMI), which classify pigmentation intensity and extent (10–12). Complementary diagnostic approaches, such as photographic documentation and, in doubtful cases, histopathological evaluation, can help distinguish benign melanosis from other pigmented lesions (9, 13).

The treatment possibilities are numerous and consist of eliminating both the epithelial layer and the underlying connective epithelium to generate a new gingival epithelium devoid of melanin (14, 15). Techniques described include surgical scraping, electrosurgery, cryosurgery, and various types of lasers (e.g., Er:YAG, Nd:YAG, CO₂, diode) (7). In scientific databases, numerous articles can be found that address the different treatments available to eliminate gingival pigmentation (2, 5). However, studies evaluating the level of knowledge and diagnostic competence of dental professionals or students regarding gingival melanosis are notable scarce. The literature focuses primarily on prevalence or therapeutic procedures, with limited attention to how well dental professionals recognize, diagnose, and manage this condition (16). This gap highlights the need for educational research exploring awareness and understanding of gingival melanosis within the academic and clinical community. The possible lack of knowledge about this condition and its treatment options appeared to be an interesting aspect to investigate. Therefore, a cross-sectional observational study based on a questionnaire was conducted to explore knowledge related to gingival melanosis, including its diagnosis, etiologies, treatment, and recurrence. Dentists of various specialties and fourth- and fifth-year dentistry students were asked to answer our questionnaire to evaluate the level of knowledge.

The main objective of the present study was to evaluate the level of knowledge of gingival melanosis among dentistry professionals and students. The specific objectives include evaluating the level of knowledge about the ability to diagnose the presence of gingival melanosis, the possible etiologies, the different methods to estimate the degree of melanosis, the different existing treatments and the probability of recurrence, and allowing an evaluation of the interest in training in the treatment of gingival melanosis.

## Material and method

### Study design

An observational, cross-sectional and descriptive study was carried out based on a survey on the topic of gingival melanosis, which was completed by professors and students from the European University of Valencia (UEV) (Valencia Campus, Paseo de la Alameda, 7, 46010 Valencia, Spain). The writing was carried out following the STROBE Guide. The study was approved by the Research Ethics Committee of the European University of Madrid; with code: 2023-372.

### Sample selection

The responses of the fourth- and fifth-year students of the UEV dentistry degree and of the UEV professors who graduated in dentistry in the period between February and March 2024 were included. All other students and teachers from different grades and courses were excluded. To calculate the minimum sample, the number of surveys to be applied in the study, accepting a 95% confidence interval and an alpha risk of 0.05, was 191 to achieve the necessary statistical power.

### Procedure description

The survey used in this study was specifically designed by the authors. The questionnaire was developed using the Google forms online platform and subsequently distributed by email to the graduate professors in Dentistry working at the UEV as well as to fourth- and fifth-year dentistry students enrolled at the same institution. To ensure content validity, the initial version of the questionnaire was reviewed by a panel of seven independent experts, including academic staff and clinicians representative of the target profiles. The experts assessed each item in terms of clarity, relevance, and pertinence, and provided feedback that was used to refine the final version. None of the experts participated in the subsequent data-collection phase. Reliability was preliminarily evaluated through inter-rater agreement (Cohen's Kappa coefficient) for key diagnostic items, confirming adequate consistency. Additionally, internal consistency of the final questionnaire was tested by calculating Cronbach's alpha, which demonstrated satisfactory reliability.

### Data collect

The survey was made up of 22 questions divided into 4 parts. The first part was made up of 6 questions about the sociodemographic situation of the participant. The second part consisted of 9 questions about knowledge of gingival melanosis. The third part consisted of 5 questions about existing treatments for gingival melanosis and the possibility of recurrence. The last part consisted of 2 questions about the participant's opinion on the possible impact on the patient's quality of life and whether they would be interested in training in the management of gingival melanosis. It was estimated that it takes about 5 min to complete the survey.

### Statistic analysis

Data were analyzed using IBM SPSS Statistics (version 23.0) by an independent statistician. Prior to analysis, data were screened for completeness and consistency, excluding incoherent responses. Descriptive statistics were computed for all variables. Categorical data were expressed as absolute (*n*) and relative (%) frequencies, and quantitative data as means and standard deviations.

The internal consistency of the questionnaire was verified using Cronbach's alpha. Data normality was tested with the Kolmogorov–Smirnov test. As distributions were non-normal (*p* < 0.05), non-parametric tests were applied. Group comparisons were performed using the Mann–Whitney U or Kruskal–Wallis tests for continuous variables, and the Chi-square (*χ*^2^) or Fisher's exact test for categorical variables. Effect sizes (Cohen's d or Cramer's V) were calculated to assess the magnitude of significant differences.

A *p*-value < 0.05 was considered statistically significant. Given the descriptive and comparative aims of this cross-sectional design, regression analyses were not performed.

## Results

The sample under study is made up of 201 people related to the field of dentistry, of which 65.2% are students and 34.8% are professionals from different specialties. We will now analyze each of the responses of the respondents, comparing the students' responses with those of experienced professionals (Table 1).

**Table 1:** Demographic characteristics of the study sample.

Employment status		Education level distribution		Distribution by age group (%)				
Status	(%)	Status	(%)	18–24 years	25–34 years	35–44 years	45–54 years	55 or older
Students	65.2%	PhD Student	5.4%	71.6%	28.4%	0.0%	0.0%	0.0%
		Dentistry Student	58.8%					
		Postgraduate Student	1.0%					
Professional	34.8%	Stomatologist	1.5%	4.4%	29.4%	45.6%	11.8%	8.8%
		Maxillofacial Surgeon	0.5%					
		Dentist	32.8%					

Data are expressed as percentages (%). Age group distribution refers to the proportion within each employment category.

### Ability to diagnose the presence of gingival melanosis

100% of those surveyed answered that they know the dark pigmentation on the gingiva. Furthermore, 99% know the term gingival melanosis and only 1% are unaware of it.

### Possible etiologies of gingival melanosis

7.46% answered that they do not know what factors are associated with gingival melanosis, 8.96% that it is associated with hormones, 3.48% with medications, 4.48% to metals, 7.46% to tobacco and 67.16% considered that it is associated with all the factors considered.

### Different diagnostic methodologies for gingival melanosis

Regarding the action against the detection of gingival melanosis, it was observed that 23.88% give an explanation but do not talk about the treatments, 42.79% give an explanation and talk about the treatments, 6.47% do not gives importance and do not give an explanation, 22.39% do not give an explanation unless they are asked.

There is a significantly greater statistical difference (*p* < 0.001) in the frequency of cases detected both monthly and annually by professionals compared to the frequency of cases detected by students (Table 2).

**Table 2:** Statistical analysis of the frequency of detection of gingival melanosis on monthly and annually by students and practitioners.

Detection of gingival melanosis		N	Mean	Standard deviation	Standard error	Confidence interval for the mean at 95%		Minimum	Maximum
						Lower limit	Upper limit		
Monthly frequency	Student	131	0.36	0.657	0.057	0.25	0.47	0	3
	Professional	70	1.66	3.073	0.367	0.92	2.39	0	20
Annual frequency	Student	131	0.98	1.430	0.125	0.74	1.23	0	7
	Professional	70	8.50	13.771	1.646	5.22	11.78	0	80

Furthermore, the percentage of people who answered that they do not know the Dummet-Gupta Oral Pigmentation Index (DOPI) is significantly higher in the case of students (*p* = 0.048) (Table 3). Regarding the indices of melanin pigmentation and gingival pigmentation, respectively, 92.54% and 89.05% were unaware of them.

**Table 3:** Statistical analysis of DOPI index knowledge by students and professionals.

Do you know the DOPI index?	Employment status					
	Student		Professional		Total	
	Recount	Percentage	Recount	Percentage	Recount	Percentage
I don't know it	127	96.95%	63	90.00%	190	94.53%
Yes, I know it, but I don't use it	4	3.05%	5	7.14%	9	4.48%
Yes, I use it often	0	0.00%	2	2.86%	2	1.00%

### Existing treatments and possibilities of recurrence

Within the gingival melanosis elimination treatments, there is a statistically significant difference (*p* < 0.001) in the distribution of the responses of the two groups. The percentage of students who answered “CO_2_ laser mucoabrasion” and “diode laser mucoabrasion” is significantly higher than that of experienced professionals. While the percentage of professionals who answered that all treatments are options for eliminating gingival melanosis is significantly higher than that of students (Table 4).

**Table 4:** Statistical analysis of gingival melanosis treatment options by students and professionals.

Which of the following is a treatment for removing gingival melanosis?	Employment status					
	Student		Professional		Total	
	Recount	Percentage	Recount	Percentage	Recount	Percentage
Mucoabrasion with a scalpel	2	1.53%	1	1.43%	3	1.49%
Mucoabrasion with a turbine burr	5	3.82%	4	5.71%	9	4.48%
CO_2_ laser mucoabrasion	27	20.61%	5	7.14%	32	15.92%
Diode laser mucoabrasion	37	28.24%	6	8.57%	43	21.39%
All	54	41.22%	52	74.29%	106	52.74%
I don't know	6	4.58%	2	2.86%	8	3.98%

**Figure 1:**
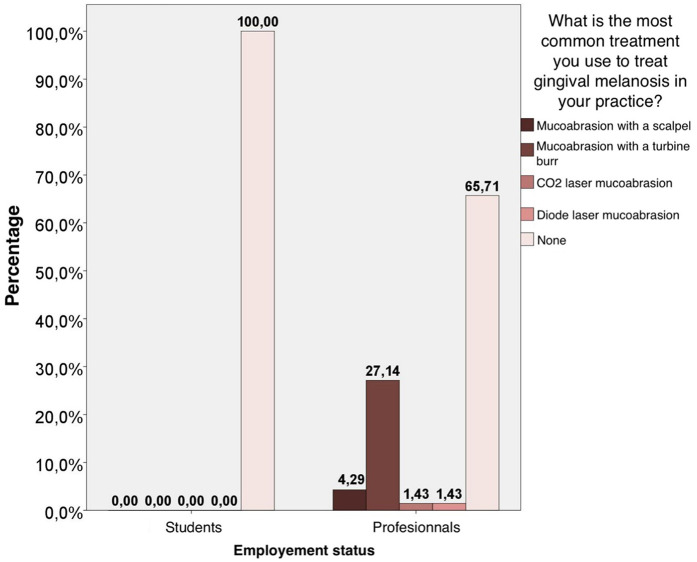
Statistical analysis of the treatment choice used in clinical practice by students and professionals.

Regarding the most used treatments, we found significant differences (*p* < 0.001) in the distribution of the responses of the two groups. The percentage of professionals who answer “mucoabrasion with a turbine burr” is significantly higher than that of students. The percentage of students who do not use any of the treatments is significantly higher than that of professionals who do not use any of these treatments; in fact, the students have never undergone treatment (Figure 1).

Regarding the effectiveness of the treatment, there are statistically significant differences (*p* < 0.001) in the distribution of the responses of the two groups. The percentage of professionals (50%) who answered “Visual clinical analysis” is significantly higher than the percentage of students (24.43%) and the percentage of students (44.27%) who answered “Both” is significantly higher than the percentage of professionals (18.57%).

Regarding knowledge about recurrence, there are statistically significant differences (*p* = 0.004) in the distribution of the responses of the two groups. The percentage of professionals who answered “always” is significantly higher than the percentage of students. The percentage of students who answered that they do not know is significantly higher than the percentage of professionals (Table 5).

**Table 5:** Statistical analysis of the opinion of students and professionals on the possibility of recurrence.

Do you believe there is recurrence in the treatment of gingival melanosis?	Employment status					
	Student		Professional		Total	
	Recount	Percentage	Recount	Percentage	Recount	Percentage
Never	4	3.05%	0	0.00%	4	1.99%
Always	8	6.11%	14	20.00%	22	10.95%
Depends on the treatment	61	46.56%	36	51.43%	97	48.26%
I didn't know	58	44.27%	20	28.57%	78	38.81%

**Figure 2:**
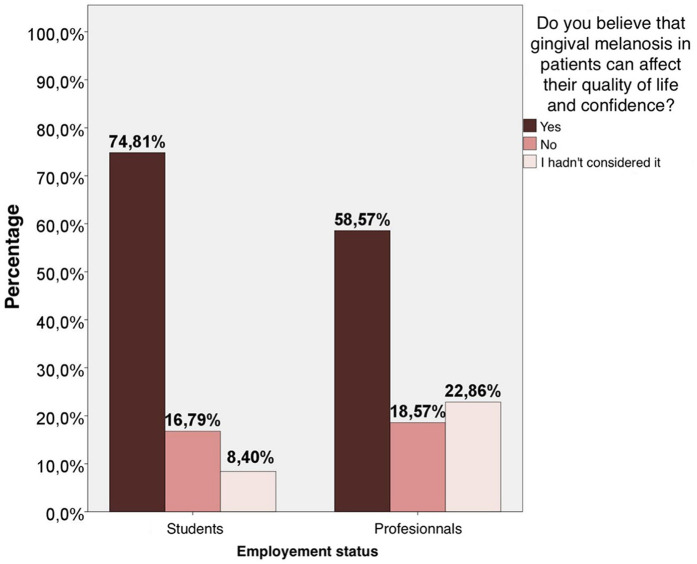
Statistical analysis of the consideration of the impact on patients’ quality of life by students and professionals.

It was observed for the consideration of the impact on quality of life that there are statistically significant differences (*p* = 0.012) in the distribution of the responses of the two groups. The percentage of students who answered “Yes” to the question is significantly higher than the percentage of professionals. The percentage of professionals who answered “I had not thought about it” to the question is significantly higher than the percentage of students (Figure 2).

### Evaluation of interest in training in the treatment of melanosis

88.06% answered “Yes, I would like to train and I would treat them”, 7.96% answered “No” and 3.98% answered “I do not think it is convenient to treat them”.

## Discussion

The present cross-sectional study was carried out with the objective of evaluating the level of knowledge of gingival melanosis among fourth- and fifth-year dental students and graduated dentists.

### Ability to diagnose the presence of gingival melanosis

In 1903, Adachi and Ramel first described gingival pigmentation (17), and in 2017, gingival color anomalies were included in gingival pigmentation according to the classification of the World Workshop on the Classification of Periodontal and Peri-Implant Diseases and Conditions (18). In the present study, all participants answered that they know the general concept of gingivalpigmentation. Furthermore, no statistically significant differences were found in the knowledge of the term gingival melanosis between both groups.

The ability to diagnose gingival melanosis is essential for dental students, as distinguishing benign physiological pigmentation from pathological or potentially malignant lesions is a fundamental component of oral diagnostic competence. Developing this diagnostic skill during undergraduate education helps prevent misdiagnosis, ensures appropriate referral and management, and promotes awareness of both functional and aesthetic aspects of gingival appearance in clinical practice (19–21).

Previous studies assessing knowledge in similar cohorts have underscored this need. For instance, cross-sectional surveys among health profession students (including non-dental disciplines) have reported gaps in understanding the etiology and management of gingival pigmentation, emphasizing the value of targeted education to enhance diagnostic accuracy and aesthetic perception. Furthermore, systematic reviews of oral pigmented lesions in syndromic patients highlight the importance of early recognition by dental trainees, as such manifestations may indicate underlying systemic conditions (9, 16, 21).

The inclusion of dental students in this survey aligns with the emphasis on early diagnostic training in oral pathology curricula. Proficiency in recognizing gingival melanosis fosters confidence during routine oral examinations and equips future practitioners to differentiate physiological pigmentation from pathological lesions—an ability regarded as essential for minimizing diagnostic errors (16, 19).

### Possible etiologies of gingival melanosis

Gingival melanosis is an alteration of multifactorial origin that can appear physiologically due to endogenous stimuli or pathologically due to exogenous stimuli (2, 4). In the present study, no statistical difference was observed between both groups in terms of knowledge of possible etiologies.

### Different diagnostic methodologies for gingival melanosis

Within the diagnosis, the part of the transmission of information to the patient can be highlighted. When we asked the participants about their way of acting when observing the presence of gingival melanosis in a patient, 42.79% answered that they give the explanation, 22.39% do not give an explanation if the patient does not ask and 6.47% do not give it importance.

Batra et al. observed that people without dental knowledge tend to notice color changes in the gingiva, especially if they are irregular, considering them unsightly (22). Therefore, the ideal would be to explain to the patient the nature of this alteration, his treatment options and allow him to decide if he wishes to receive it.

There is a statistically significant difference between the number of patients with gingival melanosis that are detected by students in the university clinic and dentists in their private clinics (between 0 and 1 vs. 1–2 in the last month; between 0 and 1 vs. 8–9 in the last year, respectively). There are several studies that count the percentage of people affected by gingival melanosis according to their ethnicity. In Europe, Hassona et al. highlighted a prevalence of 39.9% in London (23).

There are indices that allow the extent of gingival melanosis to be precisely determined (12). However, Our results reveal substantial knowledge gaps regarding gingival melanosis indices, with 92.54% of participants unaware of the melanin pigmentation index, 89.05% unfamiliar with the gingival pigmentation index, and a statistically significant difference in knowledge of the DOPI index (96.95% of students vs. 90% of faculty, *p* < 0.05). These findings highlight a limited awareness of standardized tools designed to objectively assess pigmentation extent and intensity.

Understanding and applying such indices is essential for accurate clinical documentation, esthetic evaluation, and early identification of atypical pigmentation patterns that may require further investigation. Comparable deficiencies have been described by Abdelrasoul et al. (2025), who identified inconsistencies in DOPI perception among non-dental students (16), and by Alshammari et al. (2024), who noted insufficient knowledge of baseline periodontal indices among dental trainees (24). In our cohort, the even lower familiarity observed underscores the need for integrated educational interventions—such as interprofessional and simulation-based training—to enhance diagnostic accuracy and promote standardized assessment of gingival pigmentation.

### Existing treatments and possibilities of recurrence

The choice of the type of treatment for the aesthetic removal of gingival melanosis is usually based on experience, the dentist's preference and cost (2). The different treatment options are the mucoabrasion technique with a turbine bur that was proposed in 1977 by Pérez Fernández et al. (25), mucoabrasion with a scalpel that has represented the gold standard for several years (15), mucoabrasion with a diode laser (2) or with CO_2_ laser (26). Gul et al. considered that cryosurgery and electrosurgery could represent alternatives to scalpel mucoabrasion in terms of the aesthetic results achieved (2). In the present study, 74.29% of teachers identified all treatments for depigmentation, while only 41.22% of students did, this difference being statistically significant.

The effectiveness of the treatment is verified with visual clinical analysis. But a recent study by Zahid et al. (2023) compared photographs, pre and post-depigmentation, taken with traditional and polarized techniques, and found no significant differences between the two methods (27). In the present study, a statistically significant difference is observed between the responses of the teachers and the students, the teachers (50%) considering the visual clinical analysis more effective, while the students chose the set of techniques (visual, histological and patient perception) (44.27%).

Pigmentation recurrence is the most common problem with all depigmentation procedures. In 1959, Hu proposed the “migration theory”, suggesting that there may be a process in which active melanocytes proliferate and migrate toward depigmented areas (25). Castro-Rodriguez et al. concluded that scalpel mucoabrasion had the highest frequency of recurrence, followed by diode and CO2 lasers, cryosurgery, electrosurgery and bur abrasion allowed little or even no recurrence (17). In the present study, there is a statistically significant difference between the responses of the two groups, with the number of teachers (20%) who answered that it always recurs being more important compared to the students (6.11%). But conversely, the number of students (44.47%) who responded that they did not know that gingival melanosis can recur is notable in contrast to the teachers (28.57%).

The perception that patients have of themselves is an important aspect of this alteration. Prashaanthi et al. observed that among the 150 patients affected by gingival melanosis, 12.67% limited their interactions with others, 25.33% felt uncomfortable when smiling, 29.33% had their confidence affected and 19.33% were willing to undergo depigmentation treatment (28). Likewise, Goswami et al. found that, of 300 students, 59.3% were concerned about the color of their gingiva and 54.7% were willing to undergo the procedure (29). In the present study, there is a statistically significant difference between the responses of students (74.81%) who estimated more that this alteration can have negative repercussions on the lives of patients compared to teachers (58.57%). On the contrary, it was found that the difference is significantly greater regarding the fact of raising this possibility for teachers (22.86%) than for students (8.4%).

### Evaluation of interest in training in the treatment of gingival melanosis

This study highlights the lack of knowledge about gingival melanosis, including its diagnosis, treatments, risk of recurrence and possible repercussions on patients' lives. Likewise, both dentists and future dentists can consider training to be able to offer solutions to patients.

In the present study, 88.06% of the participants are interested in training to be able to treat this alteration.

### Limitations

To our knowledge, this is the first cross-sectional observational study that compares the knowledge of students with that of dental professors on the topic of gingival melanosis. The lack of previous comparative data limits our ability to compare and extrapolate our findings to other populations. Although the minimum sample size was achieved, the total population included represents a relatively small cohort, which restricts the generalizability of the results. Additionally, the use of a self-reported questionnaire introduces a potential response bias, as participants may have overestimated or underestimated their actual level of knowledge. The absence of an objective knowledge assessment, such as case-based diagnostic tasks or image recognition tests, also limits the ability to directly measure participants' diagnostic accuracy. Another limitation relates to the participants' academic and professional profile. Dental students, by their training stage, have not yet performed surgical depigmentation procedures, which may influence their theoretical understanding. Likewise, some professionals surveyed were not specifically trained in periodontics, as the management of gingival melanosis is generally included in postgraduate programs. Therefore, it would be advisable to combine theoretical instruction with supervised clinical exposure to promote a more complete understanding of oral pigmentation disorders.

### Future lines of research

This gap in the literature highlights the need for future research on gingival melanosis in different settings. Comparing results between universities or autonomous communities could help determine whether the lack of knowledge observed at the UEV is localized or generalized. Another important aspect is the aesthetic aspect and, although it has not been evaluated in this study, it could be explored in future studies. An attractive smile involves both teeth and gingiva, however, dental training tends to focus on dental aesthetics. The lack of preparation to address gingival melanosis highlights the need to expand university training to include this aspect.

It can be concluded that it is denoted that professionals present more knowledge than students, however, a lack of general knowledge on the topic of gingival melanosis is observed. Both groups are able to detect gingival melanosis, know its scientific term, it is possible etiologies and its treatment options. However, professionals have more control over the DOPI index and the possibility of recurrence. Most participants are interested in training in the treatment of gingival melanosis. These findings suggest the need to reinforce dental curricula and continuing education programs with specific content and awareness initiatives on gingival pigmentation and its clinical management.

## Data Availability

The original contributions presented in the study are included in the article/Supplementary Material, further inquiries can be directed to the corresponding authors.

## References

[1] Shaheen RS Alsaif FM Alghofaily GA Alhumaid NS Almusallam RZ Alharthi R. The prevalence and extent of physiological and pathological gingival pigmentation in patients visiting Riyadh elm university clinics. Pak J Med Health Sci. (2021) 15(10):3039–43. 10.53350/pjmhs2115103039

[2] Gul M Hameed M Nazeer M Ghafoor R Khan F. Most effective method for the management of physiologic gingival hyperpigmentation: a systematic review and meta-analysis. J Indian Soc Periodontol. (2019) 23(3):203. 10.4103/jisp.jisp_555_18 31143000 PMC6519100

[3] Castro-Rodríguez Y. Melanosis gingival, una revisión de los criterios para el diagnóstico y tratamiento. Odontoestomatologia. (2019) 21(33):54–61. 10.22592/ode2019n33a7

[4] Osorio Ayala LD Cantos Tello PM Carvajal Endara AS. Gingival melanosis: diagnosis and therapy of its aesthetic involvement. Literature review. ODOVTOS-Int J Dent Sc. (2020):192–204. 10.15517/ijds.2021.44128

[5] Alhajj MN Alhajj WA. Prevalence of melanin pigmentation in a Yemeni population and its relation to some risk factors. Braz Dent Sci. (2020) 23(2):1–9. 10.14295/bds.2020.v23i2.1906

[6] Jain SK Shenoy N Chourasia MK Ramesh A. A comparative clinical study on surgical blade and diode Laser in the treatment of gingival melanin pigmentation. J Evol Med Dent Sci. (2021) 10(10):689–93. 10.14260/jemds/2021/148

[7] Surve P Mudda JA Patil VA Desai SR Agarwal P Mustafa M. Gingival depigmentation using surgical scalpel and sieve method of diode laser techniques - a comparative clinical intervention study. J Evol Med Dent Sci. (2020) 9(29):2063–7. 10.14260/jemds/2020/449

[8] Meleti M Vescovi P Mooi WJ van der Waal I. Pigmented lesions of the oral mucosa and perioral tissues: a flow-chart for the diagnosis and some recommendations for the management. Oral Surg Oral Med Oral Pathol Oral Radiol Endod. (2008) 105(5):606–16. 10.1016/j.tripleo.2007.07.047 18206403

[9] Abati S Sandri GF Finotello L Polizzi E. Differential diagnosis of pigmented lesions in the oral Mucosa: a clinical based overview and narrative review. Cancers (Basel). (2024) 16(13):2487. 10.3390/cancers16132487 39001549 PMC11240708

[10] Uratani AM da Cruz Perez DE Vargas PA Jorge J Lopes MA. Oral melanoma: review of theliterature. Braz J Oral Sci. (2004) 3(9):428–32. 10.20396/bjos.v3i9.8641741

[11] Dummet CO Gupta OP. Estimating the epidemiology of oral pigmentation. J Natl Med Assoc. (1964) 56(5):419–20. PMID: 14202808 PMC2610754

[12] Peeran SW Ramalingam K Peeran SA Altaher OB Alsaid FM Mugrabi MH. Gingival pigmentation index proposal of a new index with a brief review of current indices. Eur J Dent. (2014) 08(02):287–90. 10.4103/1305-7456.130640 PMC4054065 24966785

[13] El Shenawy HM Nasry SA Zaky AA Quriba MA. Treatment of gingival hyperpigmentation by diode laser for esthetical purposes. Open Access Maced J Med Sci. (2015) 3(3):447–54. 10.3889/oamjms.2015.071 27275269 PMC4877838

[14] Hassan S Dhadse P Bajaj P Subhadarsanee C. A comparison between the efficacy of scalpel and Laser procedures for treating gingival hyperpigmentation: a case report. Cureus. (2022) 14(8):e27954. 10.7759/cureus.27954 36120278 PMC9465127

[15] Muruppel AM Pai BSJ Bhat S Parker S Lynch E. Laser-assisted depigmentation—an introspection of the science, techniques, and perceptions. Dent J (Basel). (2020) 8(3):88. 10.3390/dj8030088 32781667 PMC7558501

[16] Abdelrasoul M Alofi D Teskieh H Alharbi R Algheryafi S. Comparison of knowledge and esthetic perception regarding gingival pigmentation between students from different non-dentistry health profession programs. BMC Med Educ. (2025) 25(1):1287. 10.1186/s12909-025-07868-3 41039471 PMC12492860

[17] Castro-Rodríguez Y Bravo-Castagnola F Grados-Pomarino S. Repigmentación melánica de la melanosis gingival. Revisión Sistemática. Rev Clín Periodoncia Implantol Rehabil Oral. (2016) 9(3):238–43. 10.1016/j.piro.2016.06.003

[18] Chapple ILC Mealey BL Van Dyke TE Bartold PM Dommisch H Eickholz P Periodontal health and gingival diseases and conditions on an intact and a reduced periodontium: consensus report of workgroup 1 of the 2017 world workshop on the classification of periodontal and peri-implant diseases and conditions. J Periodontol. (2018) 89(S1):74–84. 10.1002/JPER.17-0719 29926944

[19] Gondak RO da Silva-Jorge R Jorge J Lopes MA Vargas PA. Oral pigmented lesions: clinicopathologic features and review of the literature. Med Oral Patol Oral Cir Bucal. (2012) 17(6):e919–24. 10.4317/medoral.17679 22549672 PMC3505710

[20] Powell JP Cummings CW. Melanoma and the differential diagnosis of oral pigmented lesions. Laryngoscope. (1978) 88(8):1252–65. 10.1288/00005537-197808000-00006 672358

[21] Gonçalves IMF Gomes DQC Pereira JV Nonaka CFW Alves PM. Clinical and histopathological study of the oral multifocal melanoacanthoma: a case report. J Clin Exp Dent. (2019) 11(4):e391–4. 10.4317/jced.55344 31110620 PMC6522109

[22] Batra P Daing A Azam I Miglani R Bhardwaj A. Impact of altered gingival characteristics on smile esthetics: laypersons’ perspectives by Q sort methodology. Am J Orthod Dentofacial Orthop. (2018) 154(1):82–90.e2. 10.1016/j.ajodo.2017.12.010 29957325

[23] Hassona Y Sawair F Al-karadsheh O Scully C. Prevalence and clinical features of pigmented oral lesions. Int J Dermatol. (2016) 55(9):1005–13. 10.1111/ijd.13133 26711197

[24] Smith CS English S Quock RL Krzanowski S. From dialogue to action: assessing best practices and actionable steps for oral health professions education clinical and learning environments. J Dent Educ. (2025) 89(5):787–97. 10.1002/jdd.13745 40422323 PMC12108220

[25] Castro Rodríguez Y Grados-Pomarino S. Tratamiento de la melanosis gingival y evaluación de la repigmentación melánica. Reevaluación clínica al cabo de 2 años. Rev Clín Periodoncia Implantol Rehabil Oral. (2015) 8(2):139–43. 10.1016/j.piro.2015.06.001

[26] Esen E Haytac MC Öz İA Erdoğan Ö Karsli ED. Gingival melanin pigmentation and its treatment with the CO2 laser. Oral Surg Oral Med Oral Pathol Oral Radiol Endod. (2004) 98(5):522–7. 10.1016/j.tripleo.2004.02.059 15529122

[27] Zahid TM Natto ZS. Validity and reliability of polarized vs non-polarized digital images for measuring gingival melanin pigmentation. Clin Cosmet Investig Dent. (2023) 15:189–97. 10.2147/CCIDE.S422139 37720312 PMC10504902

[28] N P. Prevalence of gingival pigmentation and its psychological effect in Chennai population. Biosci Biotechnol Res Commun. (2020) 13(8):233–8. 10.21786/bbrc/13.8/143

[29] Goswami V Menon I Singh A Pal R Sharma A Singh VR. Knowledge, attitude and perception of gingival pigmentation among students aged 18–23 years in UP, India. J Dent Spec. (2017) 5(1):49–52. 10.18231/2393-9834.2017.0011

