# Supplementary material for: Evaluation of the degree of knowledge of gingival melanosis between professionals and students: observational and cross-sectional study
Source: Front Oral Health. 2025 Nov 28;6:1707785. doi: 10.3389/froh.2025.1707785 (PMC12698591; doi:10.3389/froh.2025.1707785)
Supplement: Supplementary file 1 [file Datasheet1.pdf]

Survey on Gingival Melanosis in Dental Practice

Please answer each question by selecting the option(s) that best describe your knowledge and experience. If more than one option applies, select all that are correct. An asterisk (*) indicates a mandatory question.

Demographics

1. Do you agree to participate in this survey voluntarily? *

- ☐ Yes
- ☐ No

2. Gender:

- ☐ Male
- ☐ Female
- ☐ Other

3. Age range:

- ☐ 18–24 years
- ☐ 25–34 years
- ☐ 35–44 years
- ☐ 45–54 years
- ☐ 55 or older

4. Highest level of dental education:

- ☐ Dental student
- ☐ Dentist
- ☐ Stomatologist
- ☐ Oral and maxillofacial surgeon
- ☐ Postgraduate student
- ☐ PhD candidate
- ☐ PhD (Doctorate)

5. Please indicate your specialty/discipline:

- ☐ General dentist
- ☐ Oral surgery specialist

- ☐ Stomatology
- ☐ Oral and maxillofacial surgery
- ☐ Periodontics
- ☐ Endodontics
- ☐ Esthetic/cosmetic dentistry
- ☐ Pediatric dentistry
- ☐ Orthodontics
- ☐ Prosthodontics
- ☐ Student
- ☐ Other (please specify): ______

6. Years in dental practice:

- ☐ None
- ☐ < 5 years
- ☐ 5–10 years
- ☐ 11–20 years
- ☐ > 20 years

Gingival Melanosis

7. Are you familiar with dark, brown, or black discolorations of the gingiva?

- ☐ Yes
- ☐ No

8. Are you familiar with the term “gingival melanosis”?

- ☐ Yes
- ☐ No

9. In your opinion, gingival melanosis may be associated with (select all that apply):

- ☐ Medications
- ☐ Metals
- ☐ Hormones
- ☐ Tobacco use
- ☐ All of the above
- ☐ I do not know

10. When you incidentally detect gingival melanosis during an oral examination, you:

- ☐ Do not consider it important and do not provide an explanation
- ☐ Do not explain it unless the patient asks
- ☐ Explain it but do not discuss treatment options
- ☐ Explain it and discuss treatment options for depigmentation
- ☐ Emphasize the potential pathological implications
- ☐ Emphasize the esthetic implications

11. How many patients with gingival melanosis have you seen in the last month?

    - (please enter a number): ______

12. How many patients with gingival melanosis have you seen in the last year?

    - (please enter a number): ______

13. Are you familiar with the DOPI index?

- ☐ Yes, I use it frequently
- ☐ Yes, I am familiar with it but do not use it
- ☐ I am not familiar with it

14. What does a score of 2 represent in the Melanin Pigmentation Index?

- ☐ A solitary unit of pigmentation in the interdental papilla without extension between adjacent solitary units
- ☐ A continuous ribbon-like band formed by extension between adjacent solitary units
- ☐ I do not know

15. What does a score of 2 represent in the Gingival Pigmentation Index?

- ☐ Brown to black spots or pigments
- ☐ Brown to black patches, but not diffuse pigmentation
- ☐ I do not know

Treatments for Melanosis

16. Which of the following are treatment options for eliminating gingival melanosis? (select all that apply)

- ☐ Mucoabrasion with high-speed bur

- ☐ Mucoabrasion with scalpel
- ☐ Mucoabrasion with diode laser
- ☐ Mucoabrasion with CO_2 laser
- ☐ All of the above
- ☐ Other (please specify): ______

17. Which treatment do you most frequently use to manage gingival melanosis in your practice?

- ☐ Mucoabrasion with high-speed bur
- ☐ Mucoabrasion with scalpel
- ☐ Mucoabrasion with diode laser
- ☐ Mucoabrasion with CO_2 laser
- ☐ All of the above
- ☐ Other (please specify): ______

18. Regarding the previous question, how many patients with gingival melanosis have you treated for depigmentation?

    - (please enter a number): ______

19. In your experience, does depigmentation treatment for gingival melanosis recur?

- ☐ Never
- ☐ Always
- ☐ It depends on the treatment modality
- ☐ I was not aware of recurrence

20. When have you most commonly observed recurrence after treatment?

- ☐ 6 months
- ☐ 1 year
- ☐ 2 years
- ☐ Other (please specify a time frame): ______

21. Have you used laser therapy to eliminate gingival melanosis?

- ☐ Yes, sometimes
- ☐ Yes, always
- ☐ No, never
- ☐ No, but I would like to

22. If you have used laser therapy, please indicate the laser type:

- (please specify): ______

23. How do you assess the effectiveness of treatment for gingival melanosis?

- ☐ Visual clinical assessment
- ☐ Histological assessment
- ☐ Patient-reported perception (subjective assessment)
- ☐ Both of the above
- ☐ None of the above

Dentists' Opinions on Melanosis and Patients' Quality of Life

24. Do you consider that gingival melanosis can affect a patient's quality of life and self-confidence?

- ☐ Yes
- ☐ No
- ☐ I had not considered this

25. If you received training in gingival melanosis and its treatments, would you feel confident performing the appropriate procedures for your patients?

- ☐ Yes — I would like to receive training and would treat these cases
- ☐ No
- ☐ I do not consider it appropriate to treat them
